# Three brief pieces of statistical advice for medical peer reviewers

**DOI:** 10.1111/eci.13171

**Published:** 2019-10-29

**Authors:** Harald Heinzl, Axel Benner

**Affiliations:** ^1^ Section for Clinical Biometrics Center for Medical Statistics, Informatics, and Intelligent Systems Medical University of Vienna Vienna Austria; ^2^ Division of Biostatistics German Cancer Research Center (DKFZ) Heidelberg Germany

1

The scientific peer review of medical research papers often entails the assessment of statistical results. Medical reviewers may sometimes feel overburdened by a task for which they are usually not specifically trained for. We offer three pieces of non‐technical statistical advice.

## 1 BE SELF‐CONFIDENT!

Statistics is the natural mean to communicate empirical research results in an easy to understand and correct manner, yet statistical knowledge of medical reviewers may be limited. Being self‐confident means to be aware of these limitations and avoid two common pitfalls. The first pitfall occurs, when reviewers let themselves dazzle by statistical techniques unknown to them and the related technical jargon; the second pitfall crops up when overconfident reviewers try to interfere in complex and well‐considered statistical analyses.

There is no need to comprehend the mathematical details of a statistical technique; however, it is crucial to understand its purpose and main assumptions, otherwise the results presented in the paper cannot be sensibly assessed and interpreted in a medical context. If the authors use a statistical technique you are not aware of, then probably many future readers will also not be aware of it. Ask the authors to provide references where the method is explained in a nontechnical way; if such introductory textbooks or tutorial papers do not exist yet, then the authors themselves should be obliged to produce a brief introductory guide and make it available either as an appendix to the paper or as (electronically available) supplementary material.

## 2 A STATISTICIAN IN THE AUTHORS LIST IS NO GUARANTEE FOR THE STATISTICAL CONTENT TO BE ACCEPTABLE; EVEN LESS WHEN A STATISTICIAN IS MENTIONED IN THE ACKNOWLEDGEMENT SECTION

We all hope that professional blunders by statisticians occur rarely, but we can never rule them out completely. But besides that, even formally correct statistical analyses may be erroneously represented in the paper leading to flawed and misguided medical statements and conclusions. The reasons are multifaceted and from time to time even of a human nature. An example for the latter is the error sometimes committed during statistical consulting when the right answer is given to the wrong problem.^1^ In addition, the occasionally difficult social position of a medical layperson within a team of physicians at a medical university or research institution should not be underestimated. A statistician is usually expected to promote the publication of a research paper; serious statistical concerns are often ill received by some team members, in particular, when these concerns seem to “endanger the success” of the whole project.

The difficult‐social‐position argument also strongly speaks against a common custom among some medical reviewers: Whenever they suppose a “statistical or data problem” in a paper without a responsible statistician, they suggest that the authors bring in a statistical expert. Once a statistician is co‐authoring the revised paper (or at least is mentioned in the acknowledgement section), the paper's statistical quality is considered to be in good order now. Yet this may occasionally be an erroneous belief: if the project contains an uncorrectable fundamental flaw from the beginning, then a sophisticated statistical analysis can only conceal the problem or make it appear negligible, but it cannot resolve it. Needless to say, both demanding and performing such an analysis are not compatible with scientific standards.

## 3 IF NECESSARY—ACT RESOLUTELY!

For a reviewer, there is no need to be too meticulous. Yet, if you have the impression that reporting and interpretation of statistical results in the paper is barely comprehensible, nebulous or even misleading, then act resolutely in the interest of scientific integrity and the future readers of the journal you are reviewing for. Hence, if you are convinced that the study design or the data are fundamentally flawed, why not simply reject the paper? Otherwise, ask the authors detailed questions, express your concerns to them and strongly recommend that appropriate reporting guidelines are followed.^2^, ^3^ In some cases, it may even seem reasonable to suggest to the editors that an additional statistical review is obtained.

## CONFLICTS OF INTEREST

None.
